# Assessing the Representativeness of Population-Sampled Health Surveys Through Linkage to Administrative Data on Alcohol-Related Outcomes

**DOI:** 10.1093/aje/kwu207

**Published:** 2014-09-16

**Authors:** Emma Gorman, Alastair H. Leyland, Gerry McCartney, Ian R. White, Srinivasa Vittal Katikireddi, Lisa Rutherford, Lesley Graham, Linsay Gray

**Keywords:** alcohol-related harm, bias, health surveys, nonresponse, record linkage, Scotland

## Abstract

Health surveys are an important resource for monitoring population health, but selective nonresponse may impede valid inference. This study aimed to assess nonresponse bias in a population-sampled health survey in Scotland, with a focus on alcohol-related outcomes. Nonresponse bias was assessed by examining whether rates of alcohol-related harm (i.e., hospitalization or death) and all-cause mortality among respondents to the Scottish Health Surveys (from 1995 to 2010) were equivalent to those in the general population, and whether the extent of any bias varied according to sociodemographic attributes or over time. Data from consenting respondents (aged 20–64 years) to 6 Scottish Health Surveys were confidentially linked to death and hospitalization records and compared with general population counterparts. Directly age-standardized incidence rates of alcohol-related harm and all-cause mortality were lower among Scottish Health Survey respondents compared with the general population. For all years combined, the survey-to-population rate ratios were 0.69 (95% confidence interval: 0.61, 0.76) for the incidence of alcohol-related harm and 0.89 (95% confidence interval: 0.83, 0.96) for all-cause mortality. Bias was more pronounced among persons residing in more deprived areas; limited evidence was found for regional or temporal variation. This suggests that corresponding underestimation of population rates of alcohol consumption is likely to be socially patterned.

Population-sampled health surveys perform a vital role in shaping the development, implementation, and evaluation of public health policy and practice. These surveys are frequently used to describe health behaviors and outcomes for monitoring population trends, assessing progress toward national health targets, and informing the allocation of health service resources. However, inference drawn from health surveys is valid only under certain conditions, with bias arising from self-reports and nonresponse often being problematic (1). The latter may be increasingly salient, because many surveys are facing declining participation levels (2–4). Low participation is likely to lead to a nonrepresentative sample if those who respond differ systematically from those who do not, although this is not an inevitable corollary of nonresponse (5). It is largely this potential for bias that generates interest in understanding the consequences of nonresponse: respondents typically differ from their nonresponding counterparts. For example, they are often more affluent (6, 7) and have distinct demographic characteristics (8–10). Nonrespondents also tend to have different patterns of health-related behaviors, most commonly engaging in riskier health behaviors (11, 12), and they tend to experience poorer health outcomes (9, 13).

The implications of nonresponse depend on the outcome or association under examination. The validity of survey estimates of alcohol consumption attracts particular scrutiny, because hazardous and harmful drinkers may be difficult to contact and locate. They also may be more likely to reside outside the typical survey sampling frame, and those who refuse to participate may have different consumption patterns again. Nonresponse generally does not distort comparisons between subgroups of participants, for example, in survey estimates of socioeconomic health disparities (7, 12, 14). However, this is not always the case (13, 15), and little is known about the potential impact on estimates of social patterning of alcohol-related outcomes in particular. Understanding trends over time can also be important if varying response levels are associated with changing nonresponse bias.

The objective of this study was to assess the magnitude and patterning of nonresponse bias with a focus on alcohol-related outcomes in a series of cross-sectional health surveys in Scotland. One means of assessing bias resulting from nonresponse is to compare respondent characteristics and outcomes with those of the population to which we are attempting to generalize. Direct comparison is possible in countries where samples are drawn from an individual-level population register with unique person identification and comprehensive linkage. This allows us to explicitly identify sociodemographic characteristics and selected morbidities for all sampled individuals regardless of response status (7, 12, 16). However, such enhanced sampling frames are generally restricted to countries operating national registers (mainly in the Nordic region); in other countries, an alternative approach is needed to make comparisons between respondents and nonrespondents. We exploited record linkage of morbidity and mortality data to compare alcohol-related harm and all-cause mortality outcomes in survey respondents with contemporaneous data on the general population of Scotland. We aimed first to quantify differences in alcohol-related harm and all-cause mortality between consenting survey respondents and the general population of Scotland; second, to explore whether these differences varied geographically or by area-based levels of deprivation; and third, to assess whether the magnitude of these differences changed over time.

## METHODS

### Data

#### Scottish Health Surveys

The Scottish Health Surveys (SHeS) are a series of stratified, cluster-sampled, cross-sectional surveys designed to measure the health of a representative sample of the Scottish population living in private households (17). We used data from the surveys conducted in 1995, 1998, 2003, 2008, 2009, and 2010 (household response proportions of 81%–63%; adult response proportions of 84%–55% (18), Table 1). The surveys include detailed information on both somatic and psychological morbidities and associated risk factors. Socioeconomic and geographical data allow comparisons to be made by relative deprivation and area of residence.

**Table 1.** Response Proportions and Consent to Linkage in the Scottish Health Surveys Among Men and Women Aged 20–64 Years, 1995–2010

Survey Year	Household Response Proportion, %	Adult Response Proportion, %	Proportion Consenting to Linkage, %	No. of Men	No. of Women
1995	81	84	93	3,118	3,867
1998	77	76	92	2,944	3,674
2003	67	60	91	2,353	3,028
2008	61	54	86	1,683	2,234
2009	64	56	85	1,944	2,647
2010	63	55	86	1,894	2,571

#### Area deprivation and geographical measures

Available measures of deprivation include the Carstairs and Morris 2001 area deprivation score (19), which is a measure of small-area material disadvantage, in the 1995 and 1998 surveys and the Scottish Index of Multiple Deprivation (SIMD) (20) from the 2003 survey onward. The Carstairs and Morris 2001 measure combines information on household overcrowding, unemployment among men, occupational social class, and whether a household owns a car to reflect access of small-area populations to material resources (19). The SIMD measures multiple facets of deprivation (e.g., income, employment, health, education, skills and training, housing, and geographic access to basic services) at “data zone” level, such that relative deprivation can be assessed. Data zones are small-area geographical units, with population sizes ranging from approximately 500 to 1,000 household residents (median population of 750 (21)). There are 6,505 such data zones in Scotland. Broader geographical information is based on National Health Service (NHS) Health Boards, which are aggregated into 7 larger Health Board regions (22). In 2006, the administrative NHS Health Board boundaries were altered in a manner that prevents direct comparison with earlier time periods.

#### General population demographic data

To construct general population data comparable with each SHeS survey, we used data zone–level midyear population estimates from the National Records of Scotland (NRS) stratified by sex and age group at each survey year. Data zone–level population estimates were not available for mid-1995, however, so mid-1996 population estimates were used. NHS Health Board and small-area deprivation data can be mapped to the data zone geography, such that baseline midyear population counts in each survey year can then be aggregated by sex, age group, Health Board region, and area deprivation quintile consistent with the data available in the SHeS.

#### Morbidity and mortality records

The Scottish Morbidity Records (SMR) are hospital records drawn from routinely collected administrative NHS data across Scotland, detailing demographic and socioeconomic information (e.g., age, sex, Health Board area of residence, and quintile of deprivation), episode management, and clinical information (23). Ethical approval for the use of these data was given by the NHS Multicentre Research Ethics Committee and Privacy Advisory Committee to the Board of NHS National Services Scotland and Registrar General (Edinburgh, Scotland). All inpatient and day cases discharged from specialities other than maternity, neonatal, and geriatric long-stay specialties with alcohol-related diagnoses in any diagnostic position were considered. The SMR records have been found to be approximately 90% accurate in identifying the correct diagnosis (24) and approximately 99% complete (25). Mortality data collected by the NRS have also been linked to the SMR records and survey records of consenting respondents. We considered deaths for which alcohol was deemed to be the primary cause. Hospitalization and death records were available from 1981 to the end of 2011. We used *International Classification of Diseases, Ninth Edition*, and the *International Classification of Diseases, Tenth Edition*, codes to classify diagnoses and causes of death. The codes used to define alcohol-related hospital episodes and deaths appear in Web Appendix 1, available at http://aje.oxfordjournals.org/.

#### Construction of the analytical sample

Individual-level data from the SHeS have been confidentially linked to routinely collected hospital admission records and mortality data (17) using a probabilistic matching algorithm (26). The linkage consent rate was 89% over all 6 surveys, ranging from 93% in 1995 to 86% in 2010. The sample was restricted to consenting respondents aged 20–64 years, because this age range was available in all survey years.

To construct the denominator for the general population, we aggregated baseline population counts of individuals aged 20–64 years from the NRS midyear population estimates in 1996, 1998, 2003, 2008, 2009, and 2010 by age group, sex, Health Board region, and deprivation quintile. Follow-up was measured from a proxy interview date of July 1 in each of these years until the end of June 2011. Aggregate person-years of exposure in the absence of any event in each sociodemographic group can then be inferred from this baseline count. To identify the number and timing of events, we drew numerator data for the population from the SMR and NRS records. The day of occurrence of each event is recorded, such that time-to-event from proxy interview date can be ascertained, allowing person-time in each sociodemographic group to be censored after the event of interest, death from any cause, or the end of the follow-up period, whichever occurs first. The sum of censored person-time identified from the hospital and death registers within each sociodemographic group was removed from the aggregate person-years of follow-up within each sociodemographic group (inferred from the full baseline population counts) to obtain accurate exposure time in each sociodemographic group. The hospital admission and death records span 1981 to the end of 2011, allowing identification of respondents with preexisting alcohol-related morbidities in both the general population and among survey respondents. In calculating incidence rates, we confined the numerator data to first-ever events; accordingly, the contribution of individuals with any preexisting alcohol-related morbidities in each sociodemographic group was removed from the baseline count and associated person-time-at-risk.

**Figure 1.**
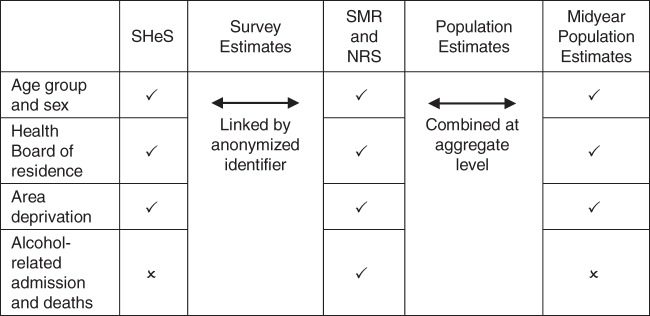
Availability of variables within data sources used to construct 2 samples for comparison. Check marks indicate that data were available, and x's indicate that they were not available. Age group and sex were recorded at baseline survey year, either as reported in the Scottish Health Surveys (SHeS); at the midpoint of the survey fieldwork period for Scottish Morbidity Records (SMR) and National Records of Scotland (NRS) data; or as recorded in midyear population estimates. Health Board area of residence (i.e., 1 of the 7 areas that comprise the National Health Service Health Board regions (22)) was measured at baseline survey year for SHeS respondents; at the time of registration for SMR and NRS data; or as recorded in midyear population estimates. The area deprivation measures used are the Carstairs and Morris area deprivation score (19) in 1995 and 1998; the Scottish Index of Multiple Deprivation (20) in 2004; and the Scottish Index of Multiple Deprivation 2012 in 2008–2010. The Carstairs and Morris score and SIMD were recorded as of the time of registration for SMR and NRS data.

Numerator and denominator data were categorized into cells according to 5-year age group, sex, quintile of deprivation, Health Board region, and data source (i.e., survey respondents or general population). The survey data were weighted using previously derived survey weights that adjust for differential probability of selection due to sampling design and differential nonresponse as a function of the sociodemographic attributes of age group, sex, and region in the 1995 (27), 1998 (28), and 2003 (29) surveys, and more recently (from 2008), whether a household is located in the most deprived 15% of data zones as measured by the SIMD (30). The data sources we used are summarized in Figure 1.

### Statistical methodology

We compared the following 2 outcomes between the SHeS respondents and the general population of Scotland: time to first occurrence of an alcohol-related harm (first-ever alcohol-related hospitalization or alcohol-related death) and time to death from any cause. Robust Poisson models were used (31) to allow for extra-Poisson variation associated with the use of survey weights, and 4 models were explored for each outcome. The purpose of the first model, model 1, was to quantify the ratio of the rate of each outcome in the survey participants compared with the rates in the general population. This was fitted separately by sex and time period (2008, 2009, and 2010 data were pooled for all analyses because of small numbers of events) and related the outcome to a set of age-group indicators and an indicator that assumed the value 1 for the survey data and 0 for the general population data. An offset was included to account for varying exposure time. Age-specific rates of the outcome within the survey and population data were generated and directly age standardized to the 1976 European Standard Population (32). To assess the magnitude of nonresponse bias, we calculated the ratio of the standardized survey rate to the standardized population rate and 95% confidence intervals.

Model 1 was then modified to further explore variation in any nonresponse bias. Model 2 pooled the data across all time periods and augmented the specification given in model 1 with main effects and an interaction between time period and data source to assess whether the age-adjusted nonresponse differential varied over time. Model 2 was fitted by sex, because there was some evidence that the level of nonresponse bias over all survey years combined, as well as the change over time, differed by sex. Model 3 added a deprivation quintile covariate and an interaction between deprivation and data source to model 2. Separately, model 4 added an indicator for each Health Board region and the interaction between these and the data source. We present *P* values from a 2-sided Wald test assessing whether the Health Board data source interaction terms are jointly equal to 0. The exponentiated coefficient (i.e., rate ratio (RR)) on the interaction term may be interpreted as a ratio of the survey-to-population rate ratio in 1 subgroup to the survey-to-population rate ratio in the reference subgroup in the discrete case, or a ratio of 2 survey-to-population rate ratios associated with a 1-unit increase in a covariate in the continuous case (33). As a robustness check, we also repeated the above analysis for time until first event of alcohol-related harm, including in those with preexisting morbidities. Analyses were conducted using Stata/SE, version 13.1, software (StataCorp LP, College Station, Texas).

## RESULTS

Among the 3,118 men aged 20–64 years in the 1995 SHeS who consented to linkage, 205 (6.6%) were subsequently hospitalized, and 27 (0.9%) died from a primarily alcohol-related cause. For the 3,867 female respondents, these figures were 113 (2.9%) and 15 (0.4%), respectively. The corresponding population figures showed that 122,660 (8.2%) of 1,492,868 men were hospitalized, and 12,883 (0.9%) died from alcohol-related causes. For the 1,551,069 women in the general population, these figures were 53,938 (3.5%) and 5,868 (0.4%), respectively (counts for all years are presented in Web Table 1).

### Comparison of survey respondents and the general population

Table 2 describes the overall degree of nonresponse bias in the incidence of alcohol-related harm and all-cause mortality by time period and sex, reporting age-standardized incidence rates of alcohol-related harm, age-standardized rates of all-cause mortality, and the corresponding survey-to-population rate ratios for both of these outcomes. Over all time periods combined, the survey-to-population rate ratios for the incidence of alcohol-related harm were 0.65 (95% confidence interval (CI): 0.56, 0.73) among men and 0.76 (95% CI: 0.65, 0.88) among women. For all-cause mortality, these figures were 0.82 (95% CI: 0.74, 0.90) and 1.00 (95% CI: 0.89, 1.10), respectively.

**Table 2.** Rates of First-Ever Events of Alcohol-Related Harm and All-Cause Mortality Per 100,000 Person-Years at Risk Among Scottish Health Survey Respondents and the General Population of Scotland Aged 20–64 Years, 1995–2010

Year by Sex	SHeS Rate	95% CI	General Population Rate	95% CI	Rate Ratio	95% CI
*Time to First-Ever Alcohol-Related Harm*						
Men						
1995	307	248, 366	481	436, 527	0.64	0.50, 0.77
1998	324	249, 399	475	432, 518	0.68	0.51, 0.85
2003	264	187, 341	462	422, 502	0.57	0.40, 0.75
2008–2010	250	153, 347	404	380, 428	0.62	0.38, 0.86
All years	299	261, 337	463	441, 485	0.65	0.56, 0.73
Women						
1995	130	100, 159	196	182, 210	0.66	0.50, 0.82
1998	144	107, 181	199	185, 212	0.72	0.53, 0.92
2003	195	131, 258	203	188, 217	0.96	0.64, 1.28
2008–2010	184	92, 275	188	178, 198	0.98	0.49, 1.47
All years	151	129, 173	197	190, 204	0.76	0.65, 0.88
Men and women	223	201, 245	324	312, 337	0.69	0.61, 0.76
*Time to Death From Any Cause*						
Men						
1995	709	620, 797	915	847, 984	0.77	0.66, 0.89
1998	642	554, 730	766	707, 824	0.84	0.71, 0.97
2003	410	311, 509	557	514, 600	0.73	0.55, 0.92
2008–2010	315	210, 420	420	395, 445	0.75	0.50, 1.00
All years	604	551, 658	738	702, 774	0.82	0.74, 0.90
Women						
1995	539	469, 608	567	531, 603	0.95	0.82, 1.09
1998	489	405, 572	467	437, 498	1.05	0.85, 1.24
2003	290	205, 375	336	315, 358	0.86	0.60, 1.12
2008–2010	216	146, 286	256	243, 268	0.85	0.57, 1.12
All years	446	403, 489	446	427, 466	1.00	0.89, 1.10
Men and women	522	488, 557	585	563, 606	0.89	0.83, 0.96

Abbreviations: CI, confidence interval; SHeS, Scottish Health Survey.

### Variation over time

Over the survey years considered, survey-to-population rate ratios for the incidence of alcohol-related harm among men ranged from 0.57 to 0.68, whereas among women these figures were closer to 1.00. For all-cause mortality, the degree of nonresponse bias again tended to be greater among men than women (Table 2). Overall, the estimated per-year change in the magnitude of bias in the incidence of alcohol-related harm was not substantial among either men (RR = 0.99, 95% CI: 0.97, 1.02) or women (RR = 1.03*,* 95% CI: 1.00, 1.07); this was similar for all-cause mortality.

### Variation by deprivation and region

When combining data across all survey years, we found that greater nonresponse bias was associated with increased deprivation for both the incidence of alcohol-related harm (RR = 0.92, 95% CI: 0.86, 0.99) and all-cause mortality (RR = 0.96, 95% CI: 0.92, 1.01). This association was greatest in 1995 for both the incidence of alcohol-related harm (RR = 0.87, 95% CI: 0.78, 0.96) and all-cause mortality (RR = 0.92, 95% CI: 0.86, 0.99) and tended to be more pronounced among men compared with women. However, tests of interactions between data source, deprivation, and time period (or sex) did not provide strong evidence for statistical differences between these groups. For data combined across all years with consistent regional boundaries (from 1995 to 2003), model 4 showed no evidence of regional variation in nonresponse bias for alcohol-related harm among men (*P* = 0.74) or women (*P* = 0.81) or in all-cause mortality among men (*P* = 0.93) or women (*P* = 0.46).

Qualitatively similar results were found when considering time until first event of alcohol-related harm among the full sample; that is, including those with preexisting morbidities. Over all time periods combined, the survey-to-population rate ratios indicating the extent of nonresponse bias were 0.65 (95% CI: 0.58, 0.73) among men and 0.74 (95% CI: 0.64, 0.83) among women.

## DISCUSSION

Respondents to the SHeS experienced lower rates of all-cause mortality and incidence of alcohol-related harm than the general population of Scotland over 6 survey waves with varying lengths of follow-up. Overall, bias was more pronounced in estimates of alcohol-related harm compared with all-cause mortality and persisted after the application of survey weights and direct age standardization. The differential in health outcomes and behaviors between respondents and nonrespondents is well documented; however, existing evidence for an interaction between health-related nonresponse bias and socioeconomic status is mixed (7, 13). In particular, little is known about potential bias in the socioeconomic gradient in alcohol-related outcomes. The present study found the extent of bias—in both the rates of alcohol-related harm and all-cause mortality—to be greater among individuals residing in the most deprived areas, suggesting distortion in the estimated social gradient of these outcomes. Over time, the declining response levels in the SHeS surveys mirrors those experienced by many surveys internationally, but a parallel increase in nonresponse bias has not been detected. This is not entirely surprising because the response proportion alone is theoretically not an ideal proxy for nonresponse bias (34). Indeed, lower response may not necessarily predict further nonresponse bias (35, 36), and efforts to increase response rates do not guarantee improved representativeness (37).

Our findings corroborate those of prior studies: nonrespondents have been found to have elevated absolute risk of death (7, 38), a greater propensity to engage in risky health behaviors, and a greater risk of experiencing associated disorders (12, 39). In particular, individuals with more problematic alcohol consumption patterns are typically underrepresented in health surveys (6, 11, 40, 41), and higher rates of death among nonrespondents are often particularly pronounced for alcohol-related causes (9, 16). Several potential explanations exist for why bias may be greater for alcohol-related harm than for all-cause mortality. First, hazardous and harmful alcohol consumption is often stigmatized, potentially increasing the rates of refusal to participate. Second, problematic alcohol consumption may be associated with a higher likelihood of selective exclusion from the sampling frame—for instance, through homelessness (42) or incarceration (43). Third, hazardous and harmful drinking may decrease the likelihood of survey administrators being able to contact potential respondents, even when they remain in the sampling frame.

The use of record-linked survey data is eminently suitable for survey validation, because they enable direct comparison of the same outcomes between survey respondents and the general population, unlike other approaches to assessing bias, such as comparing the characteristics of early and late respondents. The data we used are of high quality (24, 25), and their repeated cross-sectional nature allows assessment of how the level of bias has changed over time. We have a rich source of longitudinal information with a relatively long period of follow-up and high linkage consent rates. Record-linked survey data bring a number of advantages but also have limitations. Those who do not consent to linkage in the SHeS may introduce bias if this group differs systematically from those who do consent. This effect may be limited, because those who do not consent comprise only 7%–14% of the survey respondents, and no statistically significant differences between these groups have been found in terms of weekly alcohol consumption or binge drinking (data not shown). The use of the SIMD index, which comprises multiple facets of deprivation, including death, suggests a degree of circularity in using this to assess differentials in health-related outcomes. Our findings are robust to using the subcomponent of the measure based only on income data.

A further consideration is that sampling coverage in population-sampled surveys is often confined to individuals living in private households, as in the SHeS. This excludes certain groups present in the general population, such as those living in communal establishments—for instance prisons, homeless hostels, and medical and long-term care establishments. Because this group experiences a systematically different rate of alcohol-related harm compared with the general population, correspondingly different outcomes in our comparisons are expected, even if the SHeS accurately represents its target population. Several aspects of our analysis are expected to lessen the impact of this differential sampling frame; confining the age range to 20–64 years excludes a large proportion of those residing in medical and long-term care institutions. Although it is difficult to measure, the proportion of the population of Scotland resident in communal establishments is likely to be small; data from the 2001 census indicate that approximately 1.7% were resident in communal establishments (44). The effect on our analysis depends on the extent of the differential rate of harm in this group. A Finnish study explicitly identified those typically excluded from survey sampling frames; the exclusion of this group reduced, but did not have a substantial impact on (maximum 4%), estimates of alcohol-related death (45). A lack of granular data—especially regarding death and morbidities—makes it difficult to harmonize the sampling frames entirely (e.g., by excluding institutionalized individuals from the general population data), but it is unlikely that such a correction would qualitatively affect our conclusions.

Migration also represents a potential caveat in this analysis. Individuals who emigrate outside of Scotland during the study period will have unknown health outcomes, whereas individuals entering Scotland may contribute outcomes but are not included in the baseline population estimate. The impact of this depends on the relative health of migrants; some studies of migration patterns in Scotland have found a “healthy migrant” effect (46). However, there is likely to be heterogeneity stemming from country of origin—for instance, recent increased immigration from central and eastern European countries with known higher rates of alcohol misuse (47) may offset the effect of healthy migrants.

This study capitalized on linkage of survey records to routinely collected health data to identify lower rates of alcohol-related harm and all-cause mortality among survey respondents compared with population counterparts in a series of health surveys in Scotland. The extent of bias was more pronounced among individuals living in the most deprived areas, and limited evidence was found for regional and temporal variations. These findings have wider implications for the accurate measurement of population-level alcohol consumption. A comparison of survey estimates of population-level alcohol consumption with per-capita figures derived from data on national alcohol sales reveals a coverage gap of up to 50% in Scotland (48). The results of this study suggest that this underestimation is likely to be explained to some extent by the lower response rate of individuals who consume alcohol most problematically, with this bias being greater in the most deprived areas. As the validity of population-sampled surveys is increasingly jeopardized through declining response levels, their ongoing value may be improved through the use of reliable auxiliary information to better understand and adjust for nonresponse bias. Post hoc survey adjustments (such as weighting or multiple imputation) based solely on demographic and socioeconomic characteristics are often insufficient to account for health-related differences between respondents and nonrespondents (49, 50) and may generate results that remain divergent from population-representative health outcomes. Insights gained from comparisons between survey respondents and the general population will expand opportunities for more informed weighting or imputation procedures, ultimately enabling the production of more accurate estimates of population-level alcohol intake used for policy planning and evaluation (18).

## Supplementary Material

Web Material

## References

[1] Stockwell T Donath S Cooper-Stanbury M Under-reporting of alcohol consumption in household surveys: a comparison of quantity-frequency, graduated-frequency and recent recall. Addiction. 2004;99 8:1024–1033 15265099 10.1111/j.1360-0443.2004.00815.x

[2] Galea S Tracy M. Participation rates in epidemiologic studies. Ann Epidemiol. 2007;17 9:643–653 17553702 10.1016/j.annepidem.2007.03.013

[3] Aromaa A Koponen P Tafforeau J Evaluation of Health Interview Surveys and Health Examination Surveys in the European Union. Eur J Public Health. 2003;13(3 suppl):67–72 14533752 10.1093/eurpub/13.suppl_1.67

[4] Tolonen H Helakorpi S Talala K 25-year trends and socio-demographic differences in response rates: Finnish Adult Health Behaviour Survey. Eur J Epidemiol. 2006;21 6:409–415 16804763 10.1007/s10654-006-9019-8

[5] Little RJ Rubin DB. Statistical Analysis with Missing Data. 2nd ed. New York, NY: Wiley; 2002

[6] Maclennan B Kypri K Langley J Non-response bias in a community survey of drinking, alcohol-related experiences and public opinion on alcohol policy. Drug Alcohol Depend. 2012;126(1-2):189–194 22677457 10.1016/j.drugalcdep.2012.05.014

[7] Harald K Salomaa V Jousilahti P Non-participation and mortality in different socioeconomic groups: the FINRISK population surveys in 1972–92. J Epidemiol Community Health. 2007;61 5:449–454 17435214 10.1136/jech.2006.049908 PMC2465683

[8] Schneider KL Clark MA Rakowski W Evaluating the impact of non-response bias in the Behavioral Risk Factor Surveillance System (BRFSS). J Epidemiol Community Health. 2012;66 4:290–295 20961872 10.1136/jech.2009.103861

[9] Goldberg M Chastang JF Leclerc A Socioeconomic, demographic, occupational, and health factors associated with participation in a long-term epidemiologic survey: a prospective study of the French GAZEL cohort and its target population. Am J Epidemiol. 2001;154 4:373–384 11495861 10.1093/aje/154.4.373

[10] Vinther-Larsen M Riegels M Rod MH The Danish Youth Cohort: characteristics of participants and non-participants and determinants of attrition. Scand J Public Health. 2010;38 6:648–656 20529967 10.1177/1403494810374222

[11] Zhao J Stockwell T Macdonald S. Non-response bias in alcohol and drug population surveys. Drug Alcohol Rev. 2009;28 6:648–657 19930019 10.1111/j.1465-3362.2009.00077.x

[12] Osler M Kriegbaum M Christensen U Rapid report on methodology: Does loss to follow-up in a cohort study bias associations between early life factors and lifestyle-related health outcomes? Ann Epidemiol. 2008;18 5:422–424 18329893 10.1016/j.annepidem.2007.12.008

[13] Lorant V Demarest S Miermans PJ Survey error in measuring socio-economic risk factors of health status: a comparison of a survey and a census. Int J Epidemiol. 2007;36 6:1292–1299 17898025 10.1093/ije/dym191

[14] Jones AM Koolman X Rice N. Health-related non-response in the British Household Panel Survey and European Community Household Panel: using inverse-probability-weighted estimators in non-linear models. J R Stat Soc Ser A Stat Soc. 2006;169 3:543–569

[15] Howe LD Tilling K Galobardes B Loss to follow-up in cohort studies: bias in estimates of socioeconomic inequalities. Epidemiology. 2013;24 1:1–9 23211345 10.1097/EDE.0b013e31827623b1 PMC5102324

[16] Tolonen H Laatikainen T Helakorpi S Marital status, educational level and household income explain part of the excess mortality of survey non-respondents. Eur J Epidemiol. 2010;25 2:69–76 19779838 10.1007/s10654-009-9389-9

[17] Gray L Batty GD Craig P Cohort profile: the Scottish Health Surveys cohort: linkage of study participants to routinely collected records for mortality, hospital discharge, cancer and offspring birth characteristics in three nationwide studies. Int J Epidemiol. 2010;39 2:345–350 19349480 10.1093/ije/dyp155 PMC2846439

[18] Gray L McCartney G White IR Use of record-linkage to handle non-response and improve alcohol consumption estimates in health survey data: a study protocol. BMJ Open. 2013;3 3:e002647 10.1136/bmjopen-2013-002647 PMC3612815 23457333

[19] McLoone P. Carstairs Scores for Scottish Postcode Sectors From the 2001 Census. Glasgow, Scotland: MRC Social and Public Health Sciences Unit; 2004

[20] Scottish Government. Scottish Index of Multiple Deprivation. Edinburgh, Scotland. http://www.scotland.gov.uk/Topics/Statistics/SIMD. Updated June 12, 2014. Accessed October 15, 2013

[21] Scottish Government. Scottish Neighbourhood Statistics Data Zones Background Information. Edinburgh, Scotland. http://www.scotland.gov.uk/Publications/2004/02/18917/33243. Published February 18, 2004. Updated April 7, 2006. Accessed June 3, 2013

[22] Bromley C Sproston K Shelton N The Scottish Health Survey 2003. Volume 4. Technical Report. Edinburgh, Scotland: The Stationery Office; 2005

[23] Information Services Division, NHS National Services Scotland. SMR Data Manual. Edinburgh, Scotland. http://www.ndc.scot.nhs.uk/Data-Dictionary/SMR-Datasets/. Accessed August 9, 2013

[24] Harley K Jones C. Quality of Scottish Morbidity Record (SMR) data. Health Bull (Edinb). 1996;54 5:410–417 8936810

[25] Information Services Division, NHS National Services Scotland. Hospital Records Data Monitoring: SMR Completeness Tables. Edinburgh, Scotland. http://www.isdscotland.org/Products-and-Services/Hospital-Records-Data-Monitoring/SMR-Completeness/. Accessed June 12, 2013

[26] Fleming M Kirby B Penny KI. Record linkage in Scotland and its applications to health research. J Clin Nurs. 2012;21(19-20):2711–2721 22985317 10.1111/j.1365-2702.2011.04021.x

[27] Dong W Erens B, eds. Scotland's Health: Scottish Health Survey 1995. 2 Volumes. Edinburgh, Scotland: The Stationery Office; 1997

[28] Shaw A McMunn A Field J The Scottish Health Survey 1998. 2 Volumes. Edinburgh, Scotland: The Stationery Office; 2000

[29] Bromley C Sprogston K Shelton N The Scottish Health Survey 2003. 4 Volumes. Edinburgh, Scotland: The Stationery Office; 2005

[30] Bromley C Bradshaw P Given L. Volume 2: Technical Report. The Scottish Health Survey 2008. Edinburgh, Scotland: The Scottish Government Health Directorate; 2009

[31] Wooldridge JM. Econometric Analysis of Cross Section and Panel Data. Cambridge, MA: The MIT Press; 2002

[32] Waterhouse JAH Muir CS Correa P, eds. Cancer Incidence in Five Continents, Vol. III. IARC Scientific Publications No. 15. Lyon, France: International Agency for Research on Cancer; 1976

[33] Buis ML. Stata tip 87: interpretation of interactions in non-linear models. Stata J. 2010;10 2:305–308

[34] Davern M. Nonresponse rates are a problematic indicator of nonresponse bias in survey research. Health Serv Res. 2013;48 3:905–912 23656501 10.1111/1475-6773.12070 PMC3681235

[35] Groves RM Peytcheva E. The impact of nonresponse rates on nonresponse bias: a meta-analysis. Public Opin Q. 2008;72 2:167–189

[36] Groves RM. Nonresponse rates and nonresponse bias in household surveys. Public Opin Q. 2006;70 5:646–675

[37] Christensen AI Ekholm O Kristensen PL The effect of multiple reminders on response patterns in a Danish health survey [published online ahead of print May 22, 2014]. Eur J Public Health. (doi:10.1093/eurpub/cku057) 10.1093/eurpub/cku057 24855288

[38] Hockey R Tooth L Dobson A. Relative survival: a useful tool to assess generalisability in longitudinal studies of health in older persons. Emerg Themes Epidemiol. 2011;8 1:3 21294918 10.1186/1742-7622-8-3 PMC3039541

[39] Knudsen AK Hotopf M Skogen JC The health status of nonparticipants in a population-based health study: the Hordaland Health Study. Am J Epidemiol. 2010;172 11:1306–1314 20843863 10.1093/aje/kwq257

[40] Meiklejohn J Connor J Kypri K. The effect of low survey response rates on estimates of alcohol consumption in a general population survey. PLoS One. 2012;7 4:e35527 22532858 10.1371/journal.pone.0035527 PMC3332039

[41] Ahacic K Kåreholt I Helgason AR Non-response bias and hazardous alcohol use in relation to previous alcohol-related hospitalization: comparing survey responses with population data. Subst Abuse Treat Prev Policy. 2013;8:10 23497679 10.1186/1747-597X-8-10 PMC3599287

[42] Gilchrist G Morrison DS. Prevalence of alcohol related brain damage among homeless hostel dwellers in Glasgow. Eur J Public Health. 2005;15 6:587–588 16162595 10.1093/eurpub/cki036

[43] Graham L Heller-Murphy S Aitken L Alcohol problems in a remand Scottish prisoner population. Int J Prison Health. 2012;8 2:51–59 10.1108/17449201211277174 25758016

[44] National Records of Scotland. Scotland's Census Results Online. Edinburgh, Scotland: National Records of Scotland. http://www.scrol.gov.uk/scrol/browser/profile.jsp. Updated May 2, 2003. Accessed August 27, 2013

[45] Mäkelä P Huhtanen P. The effect of survey sampling frame on coverage: the level of and changes in alcohol-related mortality in Finland as a test case. Addiction. 2010;105 11:1935–1941 21040059 10.1111/j.1360-0443.2010.03069.x PMC3058593

[46] Popham F Boyle PJ. Is there a ‘Scottish effect’ for mortality? Prospective observational study of census linkage studies. J Public Health (Oxf). 2011;33 3:453–458 21493620 10.1093/pubmed/fdr023

[47] Popova S Rehm J Patra J Comparing alcohol consumption in central and eastern Europe to other European countries. Alcohol Alcohol. 2007;42 5:465–473 17287207 10.1093/alcalc/agl124

[48] Beeston C Reid G Robinson M Monitoring and Evaluating Scotland's Alcohol Strategy. Third Annual Report. Edinburgh, Scotland: NHS Health Scotland; 2013

[49] Frankel MR Battaglia MP Balluz L When data are not missing at random: implications for measuring health conditions in the Behavioral Risk Factor Surveillance System. BMJ Open. 2012;2 4: e000696 10.1136/bmjopen-2011-000696 PMC3400062 22798250

[50] Santin G Geoffroy B Bénézet L In an occupational health surveillance study, auxiliary data from administrative health and occupational databases effectively corrected for nonresponse. J Clin Epidemiol. 2014;67 6:722–730 24491792 10.1016/j.jclinepi.2013.10.017

