# Supplementary material for: Assessing the Representativeness of Population-Sampled Health Surveys Through Linkage to Administrative Data on Alcohol-Related Outcomes
Source: Am J Epidemiol. 2014 Sep 16;180(9):941–8. doi: 10.1093/aje/kwu207 (PMC4207717; doi:10.1093/aje/kwu207)
Supplement: Web Material [file supp_180_9_941__index.html]

Assessing the Representativeness of Population-Sampled Health Surveys Through Linkage to Administrative Data on Alcohol-Related Outcomes — Web Material 

# Assessing the Representativeness of Population-Sampled Health Surveys Through Linkage to Administrative Data on Alcohol-Related Outcomes

## Web Material

Web Material

**Files in this Data Supplement:**

- Web Material - Docx file
