# Supplementary material for: Assessing the Representativeness of Population-Sampled Health Surveys Through Linkage to Administrative Data on Alcohol-Related Outcomes
Source: Am J Epidemiol. 2014 Sep 16;180(9):941–8. doi: 10.1093/aje/kwu207 (PMC4207717; doi:10.1093/aje/kwu207)
Supplement: Web Material [file supp_kwu207_kwu207supp.docx]

**Web Appendix 1.** *International Classification of Diseases, Ninth Edition*, and the *International Classification of Diseases, Tenth Edition*, codes used to classify alcohol-related hospital episodes and alcohol-related deaths.

**Alcohol-related hospital episodes**

|  |  |
| --- | --- |
| *Prior to 01/04/1996* |  |
| ICD-9 codes hospital episodes | 2651, 3039, 3050, 2918, 2910, 2913, 2915, 2919, 2911, 2912, 2918, 3575, 4255, 5353, 5710, 5711, 5712, 5713, 7607, 7598, 7903, 9800, 9801, 9809, E860, V57, E9473, E9509, E9809, E8600^*^, E8601^*^, E8609^*^, E9509^*^  5711^§^, 6554^§^, 2550^§^, 3594^§^, 5709^§^, 3483^§^, D3039+A3317^a^, D3039+A3344^a^ |
| *01/04/1996 onward* |  |
| ICD-10 codes | F10, K70, X45, X65, Y15, Y90, Y91, E244, E512, G312, G621, G721, I426, K292, K860, O354, P043, Q860, R780, T510, T511, T519, Y573, Z502, Z714, Z721 |
| **Alcohol-related deaths** | |
|  |  |
| *Prior to 01/01/2000* |  |
| Main ICD-9 codes deaths | 4255, 5710, 5711, 5712, 5713, 5714, 5715, 5718, 5719, E860, 291, 303, 305 |
|  |  |
|  |  |
| *01/01/2000 onward* |  |
| ICD-10 codes deaths | G312, G621, I426, K292, K740, K741, K742, K746, K860, F10, K70, K73, X45, X65, Y15 |

The ICD codes here follow those used by Information Services Division NHS National Services Scotland (e.g. <http://www.isdscotland.org/Health-Topics/Drugs-and-Alcohol-Misuse/Publications/2014-02-25/2014-02-25-ARHS2012-13-Report.pdf>) and the National Records of Scotland (e.g. <http://www.gro-scotland.gov.uk/statistics/theme/vital-events/deaths/alcohol-related/coverage-of-stats.html>) at the time of data acquisition, for alcohol-related hospital episodes and alcohol-related deaths respectively.

Any pair of an *external alcohol code*^*^ and *external code*^§^ constitutes an alcohol-related episode.

^a^ Combination of these codes in consecutive diagnoses listed constitutes an alcohol-related episode.

**Web Table 1.** Raw (Unweighted) Counts of Baseline SHeS Respondents; General Population Estimates Aged 20 to 64; Numbers of Alcohol-related Harm and All-cause Mortality Events Occurring During Follow-up.

| Baseline survey year | Years of follow-up available | Baseline N | | N with any hospitalization due to alcohol  during follow-up | Number of alcohol-related deaths during follow-up | N experiencing either death or hospitalization due to alcohol during follow-up | Number of deaths due to any cause during follow-up | Number experiencing either death or first ever hospitalization due to alcohol during follow-up |
| --- | --- | --- | --- | --- | --- | --- | --- | --- |
| **Men** |  |  | |  |  |  |  |  |
|  |  |  | |  |  |  |  |  |
| 1995 | 16 | 1,492,868 | | 122,660 | 12,883 | 125,125 | 182,979 | 102,427 |
| 1998 | 13 | 1,480,735 | | 106,771 | 10,758 | 108,928 | 131,577 | 83,392 |
| 2003 | 8 | 1,483,986 | | 76,129 | 6,365 | 77,571 | 64,821 | 51,943 |
| 2008-2010 | 6 ^a^ | 4,632,410 | | 76,391 | 3,997 | 77,696 | 40,595 | 36,772 |
|  |  |  | |  |  |  |  |  |
| 1995 | 16 | 3,118 | | 205 | 27 | 208 | 365 | 153 |
| 1998 | 13 | 2,944 | | 194 | 26 | 196 | 282 | 138 |
| 2003 | 8 | 2,353 | | 83 | *s* | 84 | 85 | 58 |
| 2008-2010 | 6 ^a^ | 5,012 | | 69 | *s* | 70 | 40 | 30 |
|  |  |  | |  |  |  |  |  |
| **Women** |  | |  |  |  |  |  |  |
| 1995 | 16 | 1,551,069 | | 53,938 | 5,868 | 55,278 | 126,147 | 46,563 |
| 1998 | 13 | 1,544,963 | | 47,284 | 4,922 | 48,395 | 87,928 | 38,473 |
| 2003 | 8 | 1,552,642 | | 34,235 | 2,998 | 34,956 | 41,989 | 24,573 |
| 2008-2010 | 6 ^a^ | 4,813,926 | | 34,183 | 1,957 | 34,819 | 26,290 | 17,822 |
|  |  |  | |  |  |  |  |  |
| 1995 | 16 | 3,867 | | 113 | 15 | 116 | 312 | 90 |
| 1998 | 13 | 3,674 | | 105 | 14 | 109 | 246 | 84 |
| 2003 | 8 | 3,028 | | 59 | *s* | 60 | 79 | 45 |
| 2008-2010 | 6 ^a^ | 6,722 | | 44 | *s* | 44 | 35 | 22 |
|  |  |  | |  |  |  |  |  |

^a^ Combined follow-up (2010=1; 2009=2; 2008=3). *s,* suppressed due to small cell size.

SHeS; Scottish Health Surveys.
